# Semantics–Prosody Stroop Effect on English Emotion Word Processing in Chinese College Students With Trait Depression

**DOI:** 10.3389/fpsyt.2022.889476

**Published:** 2022-06-06

**Authors:** Fei Chen, Jing Lian, Gaode Zhang, Chengyu Guo

**Affiliations:** School of Foreign Languages, Hunan University, Changsha, China

**Keywords:** semantics–prosody Stroop, English, emotion word processing, trait depression, college students

## Abstract

This study explored the performance of Chinese college students with different severity of trait depression to process English emotional speech under a complete semantics–prosody Stroop effect paradigm in quiet and noisy conditions. A total of 24 college students with high-trait depression and 24 students with low-trait depression participated in this study. They were required to selectively attend to either the prosodic emotion (happy, sad) or semantic valence (positive and negative) of the English words they heard and then respond quickly. Both prosody task and semantic task were performed in quiet and noisy listening conditions. Results showed that the high-trait group reacted slower than the low-trait group in the prosody task due to their bluntness and insensitivity toward emotional processing. Besides, both groups reacted faster under the consistent situation, showing a clear congruency-induced facilitation effect and the wide existence of the Stroop effect in both tasks. Only the Stroop effect played a bigger role during emotional prosody identification in quiet condition, and the noise eliminated such an effect. For the sake of experimental design, both groups spent less time on the prosody task than the semantic task regardless of consistency in all listening conditions, indicating the friendliness of basic emotion identification and the difficulty for second language learners in face of semantic judgment. These findings suggest the unneglectable effects of college students’ mood conditions and noise outside on emotion word processing.

## Introduction

Among all sources and respects of emotional communication cues, the comprehensive process of multisensory integration is typically employed to reach a locutionary conveyance. This ability to perceive and combine both linguistic messages (i.e., verbal content meaning) and paralinguistic messages (i.e., non-verbal cues by pragmatic context, body language, and tone of voice) facilitates sophisticated social interaction (1, 2). Yet, the co-occurring semantic meaning and emotional cues in utterances simultaneously are not always presented in a consistent state, and the very discrepant messages combined may lead to delays or even challenges to a correct interpretation of true emotional expression (3–6).

As two prime channels for emotional speech interaction, verbal content and prosodic information mainly bridge the daily communication linguistically and emotionally. The general semantic meaning of speech enjoys the main content of emotional expression, but often the paralinguistic messages serve as completion and exterior presentation in physical forms (7). Therefore, verbal content acts as the most common means, and prosodic information is one of the most fundamental aspects of social interaction (8). Prosodic cues even present a clearer emotional tendency, particularly when verbal form representation is obstructed due to implicitness or other language barriers (9). By means of changes in pitch, loudness, speech rate, and pauses (10), emotional prosody reveals various non-verbal respects of language that make speakers convey emotional cues in conversation (11). But in real communication practice, emotional prosody can be isolated from semantic information, and in return interacts with verbal content, as a consequence of irregular verbal expression [e.g., sarcasm; (12, 13)].

Empirical research under the Stroop effect paradigm (14) examined the emotional interactions with these informative dimensions through cross-channel and cross-modal experiments (9, 15). With participants facing congruent and incongruent stimuli under different modalities, the inter-competence between linguistic information and paralinguistic information in emotional speech processing would be presented, suggesting relative dominance of either of them (16, 17). Participants performed better with congruent stimuli, while the prolonged reaction time and poorer accuracy rate were caused by specific but conflicting stimuli, which was in line with the congruence-induced facilitation effect and on the other hand, the incongruence-induced interference effect (18–20).

However, interpersonal and social interactions pose challenges for major depressive people, since major depression is closely connected with cognitive impairments in memory and executive functions (21, 22). Depression, a mood disorder marked especially by sadness, inactivity, difficulty in thinking and concentration, has increasingly become a major threat to human life and has arisen significant interest both in the pathological characteristics and the social performance of the patients (23–25). And people with the depression-related illness often display a quite fixed pattern of negative thinking about experience, values, and the whole world generally, and the correct social interaction and interpretation can be compromised (26).

As a subclinical state of depression, trait depression is the exact and frequently occurred tendency of an individual to experience depressive emotions (27). Being regarded as being below the diagnostic criteria for depression clinically, trait depression shares some similarities with depression on cognitive and physiological deficits (28), including pessimism, inferiority, loneliness, and unworthiness (27). As mentioned above, people with major depressive disorder (MDD) presented no self-positivity bias, and they even presented self-negativity bias, which connected more closely with negative information, leading to more automated processing of negative information in the environment (29–31). The lack of self-positive bias makes trait-depressive individuals make people succumb to depression disorders more easily, meaning the group of people who have not yet developed depression disorder but mostly are susceptible. The trait mirrors the long-term emotional stability of their state of mind (32). Although the introduction of various experimental designs and assessment scales availed research for MDD patients, the emotional speech processing for people with trait depression lacks solid evidence. Studies on emotional speech processing in trait-depressive people are quite scarce (33), partly due to the lack of attention on this specific group of people with mood disorders tendency, and partly due to the lack of a scales for the professional assessment of depressive state and trait (26, 34).

In view of previous studies employing the Stroop-like paradigm to investigate emotional processing, only a few of them focused on college students with trait depression. What is still worth mentioning is mainly the variants of the experimental design. First, studies on emotional speech processing exploring the interaction between word information and emotional prosody are rich. The congruency of affective prosody and word valence facilitated the emotional processing, which was corroborated by later studies (35, 36). The relatively salient role of paralinguistic prosodic information over semantics in emotion word processing was presented with both cross-channel and cross-modal behavioral evidence (9). Second, many previous studies on emotional processing performed on participants with MDD showed quite consistent results. The key role of correct interpretation of emotional signals across verbal and non-verbal channels can be worse (37, 38). The cognitive patterns displayed by MDD patients presented the impaired perception of positive cues and the enhanced attention to negative cues as well in emotional communication (39, 40). Such biased emotional perception has been attested by plenty of studies *via* face recognition (41–43) and a few studies *via* voice recognition (44). These are in accordance with findings at the neurophysiologic level presenting a reduction of activation in frontal and limbic areas in MDD patients (45, 46). Third, Gao et al. (47) presented the mechanisms of the “bilingual advantage effect” under the condition of different languages in the Stroop task. It turned out that skilled bilinguals performed better and presented stronger inhibitory control ability under the condition of the first language than monolinguals. And these bilinguals possessed better information monitoring ability and conflict resolution, which shed some light on the variants of the Stroop paradigm in terms of language capability (48).

To date, very few studies on emotional processing employed vocal speech as auditory materials to explore the performance of college students with trait depression. In the research field of psychology and sociology, the study concerning emotional conflict of college students with trait depression under the Stroop paradigm variants in the visual modality merely examined the different responses of participants under emotionally consistent and inconsistent conditions between words and facial expressions (33), showing emotional consistency effects (i.e., the fact that participants had higher accuracy and shorter response time under the word–face consistent condition). Gao et al. (33) found that the accuracy of the high trait depression group was significantly higher than that of the low trait depression group in all conditions. But the response time of the high trait depression group was significantly lower under the condition of emotional inconsistency, partly because participants tended to use a kind of processing strategy to complete the cognitive task more conveniently, according to the Emotion Infusion Theory proposed by Bower (49). Therefore, high trait-depressive participants may have a state of readiness and be able to judge the valence of emotion and face more quickly.

Within the existing literature, the studies concerning emotional prosody were examined either in MDD patients or under the simplified semantics–prosody paradigm (in lack of word valence in some experiments). Of all, the marked inclination of emotional conflicts and emotional prosody in participants with MDD seems quite certain and a truism in a general way. And studies ranging from facial signals to human voice and even cross-modal are increasingly mature and complete. Yet, research on emotional prosody in trait-depressive college students under a complete semantics–prosody Stroop effect paradigm is still quite poor. Furthermore, relevant studies were all conducted under the ideal experimental condition, rather than under background noise with ecological value. Moreover, individual differences were rarely considered as a significant factor affecting participants’ performance in certain experimental circumstances. Different levels of second language proficiency and personal state of mind are not negligible. So, this study will discuss the interaction of semantic content and emotional prosody during emotion word processing by human voice under a complete Stroop effect paradigm, with different severity of Chinese trait-depressive college students as participants, trying to explore the differences between and within groups, and then to shed light on the undiscovered land.

The current study applied the experimental protocols of Schirmer and Kotz (36) and investigated the English emotion word processing in Chinese college students with trait depression through cross-channel experiments. In terms of participants, the second language proficiency and their severity of trait depression varied more or less because of the well-known individual differences, embodying some of the individuals’ proficiency in speech perception (50). For these second language learners of English, the aural English words, to some extent, turn into a language barrier as the second language proficiency, but it is less likely to appear the ceiling effect since all English words we selected in this experiment as language materials are “everyday words” with fair verbal valence. These words were produced with happy and sad emotions, which were the two most distinguished and uncontroversial emotions shared across cultures (51, 52). Besides, participants’ long-term state of mind with depressive emotions exerts influence on the perception of emotional prosody (53). Experiment 1 employed both semantic valence judgment with and without prosody–congruency stimuli (i.e., the semantic task), and emotional prosody judgment with and without semantic–congruency stimuli (i.e., the prosody task) to explore the altered perception of speech emotions. Based on the poor performance of MDD on semantic and emotional prosody work, people with trait depression might also present prolonged response time and insensitive emotion recognition on the emotion word processing through verbal and non-verbal channels, thus less Stroop effect in semantic valence judgment. Following the same protocols, Experiment 2 stimulated a more authentic locutionary situation by means of the speech-shaped noise (i.e., an energetic environmental degradation), which added difficulties and interference in emotion word perception both linguistically and prosodically, to break the limit of the singular laboratory environment and reach conclusions with broader sense (54, 55). In this case, we hypothesized that the noisy condition might aggravate the emotional perception difficulty for the high trait-depressive group.

In a nutshell, with the second language–based and psychology-related behavioral study, we aimed to explore further the mechanisms of emotional perception in college students with trait depression specifically. By contrasts between different severity of trait depression and different levels of listening conditions, practical patterns of the Stroop-like paradigm and theoretical frameworks of emotional processing would be enriched in greater detail, which could facilitate the effective probe of nature about multiple channels of the cognitive process for clinical populations.

## Materials and Methods

### Participants

In total, 48 Chinese college student volunteers (24 men and 24 women) were recruited for this experiment, all born in China and native Mandarin speakers. Age varied from 18 to 26 years. They were graduate or undergraduate students who had English as their second language, and they have passed CET-4 (College English Test Band 4). All participants’ trait depression scores were evaluated based on the Chinese version of the State-Trait Depression Scale (ST-DEP), which was proposed by Spielberger (56) and then translated into Chinese by Lei et al. (26). With evidence of being highly valid and more focused on the assessment of cognitive and affective factors, it serves as an effective measure to distinguish between depressive state and trait (57). With a full score of 16–64, students with higher scores would be regarded as participants with high-severity trait depression and likewise, college students with lower scores would be regarded as participants with low-severity trait depression in the current study (26). Specifically, the high-trait group (*n* = 24) comprised 11 men and 13 women who scored above 40 but no more than 64 in the T-DEP, while the low-trait group (*n* = 24) contained 13 men and 11 women who scored above 16 but no more than 30.

Furthermore, all participants were tested for their English skills with the LexTALE test, an efficient vocabulary test to measure L2 language proficiency (58), and phonological short-term working memory (WM) with a digit-span test, the information held temporarily for use in immediate activities such as reasoning and decision making, which serves as a significant indicator of language learning ability (59). In addition, they fulfilled the self-rating of Emotional Intelligence Test [SREIT; (60)], a 33-item scale concerning mental representation and utilization of emotions. The demographic characteristics of participants are presented in Table 1. As displayed, there did exist significant group differences between the high trait depression group and low trait depression group in terms of T-DEP and SREIT, but not in the age, LexTALE, and WM.

**TABLE 1:** Basic information of participants.

	High-trait group (*n* = 24)		Low-trait group (*n* = 24)		
	*M*	SD	*M*	SD	*p*
Chronological age	21.96	2.07	22.25	1.87	0.611
T-DEP	44.92	4.98	25.54	4.01	<0.001***
SREIT	113.26	12.37	129.63	13.76	<0.001***
LexTALE	54.74	5.82	55.48	7.46	0.703
WM	27.42	1.72	27.01	3.39	0.595

Means (and standard deviations) of chronological age, T-DEP, SREIT, LexTALE (L2 vocabulary size), and WM for the high-trait group and low-trait group. T-DEP, Trait Depression Scale; SREIT, Self-Rating of Emotional Intelligence Test; WM, working memory. ***p < 0.001.

All participants were right-handed with normal or corrected vision, and only those who reported no history of speech, hearing impairment, no musical training, or had no experience of major depressive therapy were recruited in the current study (61). This study was approved by the (institution redacted for peer review) to ensure proper compliance with the informed consent procedure. Participants completed the informed consent at the study and got reimbursed for their participation.

### Stimuli

The stimuli we employed in the study contained 120 English words (60 verbs and 60 adjectives) carefully selected from “The Oxford 3000,” a list of the 3,000 most important words to learn in English from the Oxford English Corpus, and “The Longman Communication 3000,” a list of the 3,000 most frequent words in spoken and written English that account for 86% of the language, to avoid rare words and therefore guarantee the understandability for these second language learners of English. The whole stimulus set (American spelling) included 60 positive words and 60 negative words based on a pilot study of valence ratings obtained from four advanced English speakers and one native speaker who did not participate in either of the experiments. They were instructed to judge the semantic valence of the words in a randomized order on a 5-point scale from −2 (highly negative) to 2 (highly positive). Negative words had a mean valence of −1.43 (*SD* = 0.24), and positive words of 1.44 (*SD* = 0.35), showing no significant difference in valence strength (positive words were rated just as strong as negative words). Additionally, word frequency was counted by means of the Corpus of Contemporary American English [COCA; (62)]. As shown in Table 2, the positive words presented a similar word frequency as the negative words; positive and negative words showed comparable syllable numbers.

**TABLE 2:** Word frequency and syllable numbers of selected English words.

	Type	*M* (SD)		*M* (SD)	*p*
Word frequency	Positive	44813.18 (21884.29)	Negative	39013.72 (20774.27)	0.14
	List 1	41913.77 (21433.09)	List 2	41913.13 (21638.33)	0.99
	Adjectives	40596.93 (21929.90)	Verbs	43229.97 (21051.11)	0.50
Syllable numbers	Positive	2.03 (0.78)	Negative	1.97 (0.74)	0.63
	List 1	1.98 (0.77)	List 2	2.02 (0.75)	0.81
	Adjectives	2.07 (0.84)	Verbs	1.94 (0.66)	0.34

A Canadian male speaker (35 years old) produced all English words clearly in a quiet setting with happy and sad prosody (240 stimuli = 120 words × 2 prosodic categories), which were subsequently normalized to the same duration (1,000 ms). The pitch, however, was different between happy and sad prosodies (*p* < 0.001). Specifically, words read in happy prosody had an average pitch of 154.07 Hz (*SD* = 43.04) and words read in sad prosody of only 98.72 Hz (*SD* = 6.67), which is in line with the acoustic attributes of happy and sad utterances (63). Thus, pitch variations in accordance with the speaker’s emotion serve as assistant effects for listeners to complete the prosody-identification task (64).

Moreover, though the same words were employed as stimuli in two experiments, we added noise (SNR = 10 dB) to the audio files for Experiment 2 to create a noisy condition. The whole stimuli were divided into two lists (List 1 and List 2), with each list containing 30 positive and 30 negative words spoken by happiness and sadness prosody, conveying both semantic meaning and prosodic emotion to participants simultaneously. Thus, under different instructions of tasks, participants accordingly pay selective attention to either semantic valence information or emotional prosody information of the auditory stimuli. Notably, each list was presented under arrangement on different tasks. Therefore, half of the participants already having heard one list of words under semantic instruction would hear the other list of words under prosodic instruction and *vice versa*.

### Task and Procedures

The whole experiment was conducted in a quiet room and each participant was seated in a comfortable chair facing a computer monitor, a noise-canceling headphone, and a Chronos box [an E-Prime-based device with high accuracy of response time; (65)]. Two tasks were performed for participants to selectively attend to word valence information (semantic task: positive or negative) or emotional prosody information (prosody task: happy or sad) of auditory stimuli under corresponding instructions in quiet (Experiment 1) or noisy (Experiment 2) environment. In both experiments, instructions and auditory stimuli were presented by E-Prime [Version 3.0; (66)] on the computer, with the stimulus presentation program customized in advance. Having received the standard auditory information of English words through the noise-canceling headphone (Sennheiser HD280 Pro) binaurally at a comfortable sound intensity level (65 dB SPL), participants offered their responses by pressing the corresponding button of Chronos as quickly and as accurately as possible to indicate their judgments. The accuracy and response time recorded by Chronos would then serve as the measurement.

Participants were told to complete the practice session first as a familiarization process with four spoken words in the semantic task and prosody task, respectively, and these eight words were not used in the real experiment. After participants responded, the instant accuracy would be presented on the screen. Those who reached at least 80% accuracy would enter the formal task phase. In formal experiments, for word valence and emotional prosody in each experimental stimulus, participants were instructed to pay main attention to only one respect, though the twofold pairings with two different channels presented either congruency (positive-happy, negative-sad) or incongruency (positive-sad, negative-happy). Specifically, under the instruction of semantic information, participants would identify word valence as positive or negative while ignoring the prosody in this “semantic task.” On the other way around, in the “prosody task,” participants would judge emotional prosody as happy or sad under the instruction of prosody information while ignoring word valence. Instructions were visually presented on the computer screen to make sure participants’ full understanding of each task. Stimuli were presented in a randomized fashion within different tasks to each participant. Unlike the familiarization session, no instant feedback of accuracy would be presented on the screen to avoid the unnecessary distraction of participants, and the session would proceed to the next trial if no response was recorded within 5,000 ms.

Experiment 2 followed the same procedure of the protocols and employed the same word in the quiet environment of Experiment 1, only the speech-shaped noise at a fixed signal-to-noise ratio (SNR = 10 dB) was affiliated to create a noisy environment in Experiment 2, with the effect of energetic masking (67, 68). The SNR of 10 dB was determined based on a pilot study, which reached the lowest SNR level with a minimum accuracy of 80% in both tasks. To avoid the fatigue effect, there was a short break between two experiments, which were presented to participants in random order. The whole experiment took approximately 1 h.

### Data Analyses

For statistical analyses, a range of calculations were performed in R [Version 4.1.2; (69)]. For the collected data, 48 subjects participated in two experiments, with each experiment containing 2 tasks and each task containing 120 items, 23,040 data were obtained in total (48 × 2 × 2 × 120 = 23,040 observations). As for the preliminary data filtering process, only data with reaction time between 100 ms to 2,500 ms were counted as acceptable in the experiment to enhance data validity, since neither the excessive speed nor the noticeable delay in response time was admitted in the study. Then, we eliminated incorrect responses, and 18,809 observations were kept eventually. Besides, we also performed a log transformation to reaction time data since in many perceptual experiments response time exhibits positive skewness (70). Furthermore, to compare the inter-group difference of T-DEP scores, SREIT scores, and WM between high-trait group and low-trait group, we employed two-sample *t*-tests with the R package of ez (71).

In general, a linear mixed-effect model (LMM) was constructed using the R package of lme4 (72). Considering the huge difference between types of data, the trial number of words and digit-span scores of participants were centered and therefore reached normalization. When fitting all the LMMs in the analyses of the two identification data, “Reaction time” was counted as the dependent variable. Fixed factors: “Severity (high vs. low),” “Congruency (congruent vs. incongruent),” “Task (semantic vs. prosodic),” “Condition (quiet vs. noisy),” and all their interactions; two random factors: “Participant” and “Item,” were included in the model. And controlled co-variants were LexTALE scores, working memory, and normalized trial number. By-participant random intercepts and slopes for all possible fixed factors were included in the initial model (73), which was compared with a simplified model that excluded a specific fixed factor using the analysis of variance (ANOVA) function in lmerTest package (74). The model was fitted to optimize it. Besides, Tukey’s *post hoc* tests were employed using lsmeans packages (75) to elaborate the significant interaction effects when necessary.

## Results

Statistics suggested that the mean age of participants is 22.10 (*SD* = 1.96, range = 18–26) years, and they have received an average of 15.83 (*SD* = 1.84) years of formal education. Table 3 presents the fullest results of the LMM on these participants’ reaction time across two experiments, showing a significant four-way interaction of “Severity” × “Congruency” × “Task” × “Condition” [χ^2^ (1) = 4.45, *p* < 0.05], which was further separately analyzed under two different conditions (quiet and noisy conditions), namely, Experiment 1 and Experiment 2.

**TABLE 3:** Results of linear mixed effects model on reaction time (full presentation with results of both Experiment 1 and Experiment 2).

Effect	Chi-square	*p*
Severity	4.26	0.039*
Congruency	123.90	<0.001***
Task	69.93	<0.001***
Condition	7.96	0.005**
scale_Trial	28.57	<0.001***
scale_Digit span	0.14	0.706
LexTALE	5.21	0.022*
Severity:Congruency	0.01	0.910
Severity:Task	6.45	0.011*
Congruency:Task	9.38	0.002**
Severity:Condition	17.01	<0.001***
Congruency:Condition	0.06	0.801
Task:Condition	13.56	<0.001***
Severity:Congruency:Task	0.06	0.808
Severity:Congruency:Condition	1.14	0.285
Severity:Task:Condition	0.07	0.785
Congruency:Task:Condition	2.26	0.133
Severity:Congruency:Task:Condition	4.45	0.035*

*p < 0.05, **p < 0.01, ***p < 0.001.

### Experiment 1 (Quiet Condition)

**FIGURE 1:**
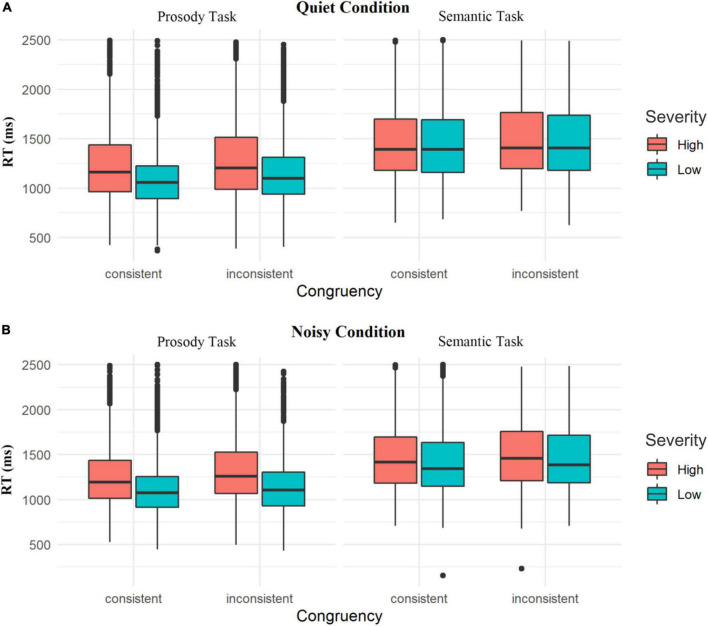
Box plots of reaction time in participants with low and high trait depression across consistency (consistent vs. inconsistent) in prosody and semantic tasks in the quiet condition **(A)** and noisy condition **(B)**.

Figure 1A shows high-severity and low-severity trait-depressive participants’ reaction time in semantic-emotion interference tasks in quiet condition. As displayed in Table 4, in the quiet condition, a significant two-way interaction of “Severity” × “Task” was found [χ^2^ (1) = 4.14, *p* < 0.05]. The following *post hoc* tests showed that when participants conducted the prosody task, high-trait group reacted slower than low-trait group (β = 0.094, *SE* = 0.047, *z* = 1.978, *p* < 0.05), but there exists no such significant difference when they conducted semantic task (β = 0.008, *SE* = 0.027, *z* = 0.278, *p* = 0.781); in general, participants reacted faster in the prosody task regardless of their trait depression scores (*p*s < 0.001).

**TABLE 4:** Linear mixed-effects model with severity, congruency, task as the fixed effects and the logarithm of reaction time as dependent variables in Experiment 1.

Effect	Chi-square	*p*
Severity	2.34	0.126
Congruency	56.36	<0.001***
Task	60.25	<0.001***
scale_Digit span	0.35	0.553
scale_Trial	14.06	<0.001***
LexTALE	5.88	0.015*
Severity:Congruency	0.63	0.427
Severity:Task	4.14	0.042*
Congruency:Task	9.82	0.002**
Severity:Congruency:Task	2.43	0.119

*p < 0.05, **p < 0.01, ***p < 0.001.

As Table 4 displays, a significant two-way interaction of “Congruency” × “Task” was found as well in the quiet condition [χ^2^ (1) = 9.82, *p* < 0.01]. More specifically, when performing both prosody (β = −0.054, *SE* = 0.007, *z* = −7.700, *p* < 0.001) and semantic (β = −0.022, *SE* = 0.007, *z* = −3.035, *p* < 0.01) tasks, both high-trait group and low-trait group spent less time under consistent situation compared with inconsistent situation. The results displayed that participants tended to be more affected by semantic congruency (or not) in the prosody task than be affected by prosody congruency (or not) in the semantic task.

There was also a clear fact that regardless of consistency or not (the Stroop effect set on the experiment), they spent less time when they conducted prosody task rather than semantic task (*p*s < 0.001).

### Experiment 2 (Noisy Condition)

Figure 1B displays high-trait and low-trait groups’ reaction time in semantic and prosody tasks under a noisy condition. As displayed in Table 5, in the noisy condition, LMM revealed a clear main effect of “Congruency” [χ^2^ (1) = 34.61, *p* < 0.001] in the noisy condition, while the two-way and three-way interactions between “Congruency” and any other factors failed to reach significance (all *p*s > 0.05). Notably, while “Congruency” and “Task” produced a two-way interaction and the Stroop effect therein made differences in the two tasks in a quiet condition, no such interaction was found in the noisy condition.

**TABLE 5:** Linear mixed-effects model with severity, congruency, task as the fixed effects and the logarithm of reaction time as dependent variables in Experiment 2.

Effect	Chi-square	*p*
Severity	4.65	0.031*
Congruency	34.61	<0.001***
Task	1539.82	<0.001***
scale_Digit span	0.00	>0.999
scale_Trial	19.97	<0.001***
LexTALE	2.07	0.150
Severity:Congruency	0.00	>0.999
Severity:Task	83.11	<0.001***
Congruency:Task	0.72	0.395
Severity:Congruency:Task	1.50	0.220

*p < 0.05, **p < 0.01, ***p < 0.001.

Besides, a significant two-way interaction of “Severity” × “Task” was detected in the noisy condition [χ^2^ (1) = 83.11, *p* < 0.001]. When participants conducted the prosody task (β = 0.126, *SE* = 0.035, *z* = 3.611, *p* < 0.001), the group difference was quite clear: high-trait group took a longer time to identify emotions expressed by noisy-interferential spoken English words than low-trait group, which closely resembled the results in the quiet condition. However, there was no such significant difference between two groups when they conducted the semantic task (β = 0.036, *SE* = 0.035, *z* = 1.035, *p* = 0.301).

Besides, in the noisy condition, both high-trait and low-trait groups took a shorter time to complete the prosody task than the semantic task (*p*s < 0.001). Primarily, these data presented that Chinese college students tended to identify emotions faster than L2 verbal content under all listening conditions.

## Discussion

So far, the question remains unresolved as to how people with a propensity to depression process emotional cues of the second language during daily communication. To fill the research gap of emotion word processing for second language learners with different severity of trait depression, the current study investigated the interaction of semantic content and emotional prosody under a complete Stroop effect paradigm by Chinese college students with trait depression in quiet and noisy environments. It was designed to address the following three research questions. First, we tried to investigate the differences between high trait-depressive group and low trait-depressive group in emotion word processing. Second, we were interested in the general mechanisms of the Stroop effect on emotion word processing in two severity of trait-depressive groups. And finally, we aimed to figure out whether any change in English emotion word processing would be posed by the influence of noise. The following discussions tried to answer these research questions based on relevant findings.

### Specific for High Trait-Depressive Group: Bluntness Toward Emotions

For the issue of Chinese college students in terms of the emotional prosody identification, results of the current study showed that in two experiments, the response time of the high-trait depression group was significantly longer than that of the low-trait depression group regardless of the congruency condition. This finding of the semantics–prosody Stroop experiment, however, is not congruent with previous findings of the word–face Stroop experiment with trait-depressive college students (33). They found that the response time of the high-trait depression group was significantly shorter than that of the low-trait depression group under the condition of emotional inconsistency. In order to explain this, the authors adopted the Affect Infusion Model (49), meaning participants took strategies in advance and processed information more conveniently, potentially accounting for this phenomenon. So, it is the earlier readiness for cognitive processing more conveniently that assisted the high-trait depression group to react faster.

The poorer performance of emotional processing in high-trait depression people is generally in line with previous studies of emotion-related judgment in patients with MDD. Previous studies presented their impaired recognition of emotions in the visual modality (i.e., facial expressions) or auditory modality (i.e., emotions are expressed vocally). They seemed to show deficits in the correct perception of affective prosody (76). And in most rating experiments, MDD tended to skew the recognition of emotional stimuli into two directions: the tendency toward negative emotional stimuli, and the bluntness of positive stimuli (38). Since trait-depressive undergraduates are associated with low heart rate variability and more specifically its parasympathetic component, which is considered a physiological index of emotion regulation capacity (77), they are less competent to regulate their emotions and reach controlled sensory processing. Participants got poor concentration toward outside information with increasing severity of depression.

Both in quiet and noisy conditions, the results of the current study showed clear contrasts of reaction time between different trait-depression groups in the prosody task, while no such significant contrast was found in the semantic task. This is plausible due to the closer connection between the prosody task and the effect on the long-term psychology of participants.

### General for Participants: Extensive Existence of Stroop Effect

Variants of Stroop effect protocols, as behavioral experiments, were considered as an exploration of the primitive operations of cognition, offering clues to the fundamental process of attention and an ideal tool for the research of automatic processing (78). Results of the current study showed the consistency facilitation effect, meaning that participants took a shorter response time under emotional consistency conditions, which is congruent with previous findings (9, 18, 19). Interestingly, the high-trait depression group lacked activated sensitivity toward emotion perception, then they might have been less affected by the change of emotional prosody when they conducted semantic tasks. However, the lack of two-way interaction of “Congruency” and “Severity” in Experiment 1, indicated that the effect of congruency of stimuli from two channels did not vary between the high-trait group and the low-trait group. Besides, the main effect of “Congruency” in Experiment 2 symbolized the “independence” of the Stroop effect from the mental state of individuals inside (i.e., participants’ trait depression severity) and environmental influences outside (i.e., quiet and noisy listening conditions). The results were roughly analogous, jointly indicating an extensive existence of the Stroop effect.

Notably, the anterior cingulate cortex and dorsolateral prefrontal cortex were reported to remain active when resolving conflict (79), indicating the brain activity in Stroop interference. And the widespread view about the Stroop effect in cognition told that mental skills (e.g., reading) are automatic once they were acquired through repetitive and extensive practice (80). Cattell (81) suggested an automatic process in the cognitive science of the Stroop effect. The automatic process was regarded as unintentional, uncontrolled, unconscious, and fast (82). Just as in the word–color experiment initially, participants in the current study could not “resist” processing word meaning in the prosodic task or pay attention to emotional prosody in the semantic task, and the interference made the difference.

### Noise in Emotion Word Processing: Masking Effect

While the Stroop effect presented high automation, it did make a varying difference between semantic task and prosody task in the quiet condition. More specifically, in Experiment 1, under the interaction of “Congruency” and “Task,” “Congruency” exerted a higher influence in the prosody task (^***^p < 0.001) than in the semantic task (^**^p < 0.01). In other words, all participants were more affected by the Stroop effect in emotion identification with the interference of English word content, which might relate to experimental design. One potential factor to account for this could be that, compared with the word valence judgment in the semantic task, identifying emotions as happy or sad in the prosody task was easier for participants, which could be proved by the reduced response time. First, we selected the two most uncontroversial emotions that share across multi-cultures as the basic emotions. Unlike other finer emotions (e.g., suspicious, surprising, sarcasm, regret), familiarity and understandability increased the response efficiency. Therefore, it is very much unlikely for participants to misinterpret them. Second, given the significant pitch difference of audio stimuli between happy tone and sad tone, there existed an obvious contrast, with happy tone much higher than sad tone (*p* < 0.001), while no such significant pitch contrasts between positive and negative words were observed (*p* = 0.808). This was not surprising since the happiness expression was always presented with explicit and unneglectable acoustic cues, such as higher pitch and quicker speed (64). Thus, participants reached faster responses within a short time. Third, although college students in this experiment have received an average of 15.83 years of education and learned English from a young age, they did not achieve perfect scores in LexTALE (*M* = 55.11). In semantic task, listening to English audio files only once and reacting within 5 s for second language learners could be a demanding task of pretty pressure, accompanied by a significant decrease of attention toward the emotional prosody of English words and a less Stroop effect. On the other hand, in the prosody task, going much easy on the emotion identification could always leave much other room for attention to verbal content, and the semantics–prosody channel congruency (positive-happy, negative-sad) or incongruency (positive-sad, negative-happy) mattered more. This aligned with the perceptual load theory: to what extent the task-irrelevant stimuli are processed is determined by whether there are spare attentional resources left when they are used to process the task-relevant stimuli (83).

However, this varying degree of Stroop effect between two tasks was not consistently observed in Experiment 2, where the listening background changed from quiet to speech-shaped noise. The interaction of “Congruency” and “Task” did not reach statistical significance in the noisy condition. Primarily, it is likely that the audio files accompanied by the noise created a relatively harsh environment for listeners to make their responses quickly. Unlike in the quiet condition where listeners could rely on the integrated prosody of words to identify emotions, they were probably forced to hear every syllable with much more effort to do the same job. And for these second language learners, mishearing only one syllable under a noisy condition could lead to loss and confusion about the lexical meaning of the whole word in the semantic task. Thus, the prosody tasks did not appear to be as easy and convenient due to the impact of noise. The increased difficulty of both tasks posed challenges for listeners to allocate their limited attention and seek the optimal solutions.

Moreover, the perception of English speech under noisy conditions occupied many more cognitive resources, including WM and inhibitory ability (84). After all, having controlled for language skills, WM, and emotional intelligence, the better inhibitory ability still predicted higher problem-solving accuracy (85). Factors such as noisy environment and other languages can adversely affect the speech perception process, leading to an increased difficulty for full understanding and a longer time to decode what was heard accurately (86). Since adverse listening conditions impair the encoding of speech signals, which means listeners have to allocate processing resources to separated aspects (87). (88, 89) also pointed out that the perceptual load of the current task participants conducted determined the allocation of cognitive resources during the process of selective attention. If the perceptual load of the current task was relatively low, and only a part of the attention resources was consumed in the process, then the extra attention resources would spare automatically to process the distractive stimuli, thus producing the distractive effect. On the contrary, if the perceptual load of the current task was high and the limited attention resources were exhausted at once, the distractible stimulation unrelated to the task could not be perceptually processed, so the distractor effect will not be produced. In all in, in the current experiment, the additional cognitive, linguistic, and perceptual resources to understand English speech in noise, heavily consumed an individual’s cognitive resources, leading to less Stroop effect.

### Limitations and Future Directions

There exist several limitations in the current study. First, the conclusions were limited to trait-depressive Chinese college students of age 18–26 years. Given the extensive distribution of this mentally impaired population of all age groups, data, and information of only a fraction of college students, with even indistinctive second language competence in hearing, might limit how we interpret the model. Whether the results mentioned above reflect the psychological features of more common people remains unclear. Thus, a larger size of participants with marked characteristics is highly needed to draw more compelling views, with the assessing scales being of high validity. Second, compared with some previous studies adopting the Stroop-like paradigm, this research only focused on the binary cross-channel contrasts of audio emotional stimuli (semantic vs. prosodic), without applying more access to communication channels and modalities (e.g., facial expressions, videos). For the experimental design, practical settings are highly feasible. For instance, more types of noises with effects of closer authentic communication simulation or even real-life environment (i.e., babble noise), diverse emotions with finer classification sharing across cultures (i.e., surprise, sarcasm), multiple approaches to keeping abreast of language and psychological research to comprehend the neurological basis (i.e., event-related potential measures). Finally, it would be beneficial for further investigations on the clinical group of MDD to apply the current findings to the clinical setting.

## Conclusion

In summary, this current study investigated psychological mechanisms of English emotion word processing under the semantics–prosody Stroop effect paradigm in quiet and noisy listening backgrounds, with Chinese college students with trait depression as participants. It was proved that the high trait depression group showed evident bluntness toward emotions compared with the low trait depression group in emotion word processing. And the widely existed Stroop effect affects the emotion word processing automatically, regardless of participants’ trait severity (i.e., high trait or low trait) and listening conditions (i.e., quiet or noisy). The results also showed that participants tended to be more affected by the Stroop effect when they conducted prosody tasks and recognized emotions than being affected in the semantic task and identified English word valence. However, such contrast was not observed with a background of speech-shaped noise, indicating the masking effect of noise on cognitive processing. Taken together, these findings provide evidence supporting the emotional processing deficit of high trait-depressive people and congruence-induced facilitation effect in widespread Stroop effect, which provide a reference on the cross-linguistic special group with multi-listening conditions for future studies and offer fairly basic evidence for clinical application of the trait depression.

## Data Availability Statement

The raw data supporting the conclusions of this article will be made available by the authors, without undue reservation.

## Ethics Statement

The studies involving human participants were reviewed and approved by the School of Foreign Languages, Hunan University. The patients/participants provided their written informed consent to participate in this study.

## Author Contributions

FC conceived and designed the study, performed the statistical analysis, and offered the financial support. JL collected and analyzed the data, and wrote the first draft of the manuscript. GZ designed the study and interpreted the data. CG participated in the statistical analysis. All authors contributed to the article and approved the submitted version.

## Conflict of Interest

The authors declare that the research was conducted in the absence of any commercial or financial relationships that could be construed as a potential conflict of interest.

## Publisher’s Note

All claims expressed in this article are solely those of the authors and do not necessarily represent those of their affiliated organizations, or those of the publisher, the editors and the reviewers. Any product that may be evaluated in this article, or claim that may be made by its manufacturer, is not guaranteed or endorsed by the publisher.
